# The Relevance of Nutrition for Pediatric Allergy and Immunity

**DOI:** 10.3390/nu15081881

**Published:** 2023-04-13

**Authors:** R. J. Joost van Neerven, Janneke Ruinemans-Koerts

**Affiliations:** 1 Cell Biology and Immunology, Wageningen University & Research, 6708 WD Wageningen, The Netherlands; 2 FrieslandCampina, 3818 LE Amersfoort, The Netherlands; 3 Laboratory of Clinical Chemistry and Hematology, Rijnstate Hospital, 6815 AD Arnhem, The Netherlands

## 1. Introduction

The development of the immune system in early life is essential to shape an immune system. The first three years (or 1000 days) of life seem to be crucial for the development of the immune system, which provides resistance to infection and cancer, and this is related to learning to tolerate non-infectious proteins it is exposed to, such as dietary proteins and other proteins, and infants should thus not develop allergies to foods and inhaled proteins. However, even though infants still have frequent respiratory and gastrointestinal infections, child mortality has been reduced significantly. Contrary to this, the prevalence of asthma, rhinitis, and food allergy has increased tremendously in recent decades. Understanding how the function and development of the immune system can be influenced in early life will hopefully help to improve protection against infection and the development of allergies in early life.

## 2. Special Issue

This Special Issue of Nutrients, entitled “The relevance of Nutrition for Pediatric Allergy and Immunity”, is an attempt to provide an overview of the current knowledge of the influence of different nutritional components both in mother and child on the prevention of infections and allergy development in children. The influence of dietary components can be provided directly to the infant by breastfeeding or early life nutrition, or indirectly, by the maternal diet (thus influencing fetal development, as well as breastmilk composition).

The influence of the immune system of pregnant women on their unborn child is regarded as the most critical phase in *the development of the fetal immune* system. To set the scene for this special issue, Warner and Warner describe the changes in the maternal immune system for protection of pregnancy and its effect on the immune system of the unborn child in early life [1]. After birth, the immune system of the neonate adapts under influence of the normal human microbiome, which is affected by many factors, such as maternal health and diet, exposure to antibiotics, mode of delivery, and breast or cow’s milk formula feeding. Nowadays, dietary composition differs largely from decades ago. Additionally, the current deficiency of specific nutrients, which affect the immune system, are associated with the increase in the prevalence of allergies. However, intervention studies, by employing single nutrient supplementation, have achieved limited proof. Two reports here describe the effect of *maternal dietary supplementation* on allergic outcomes in infants. Colquitt and colleagues performed a systematic review on such an intervention, e.g., probiotics during pregnancy [2]. They concluded that studies showed inconsistent results, from no effect to a lower incidence in children at high risk. Barman and colleagues reported, in an observational study, that higher proportions of n-6 and lower proportions of n-3 polyunsaturated fatty acids (PUFA) in the maternal diet were significantly associated with atopic eczema development in the first year of age [3]. The authors stated that their results might be influenced by the risk of residual confounding, and thus causality could not be confirmed. On the other hand, as stated by Warner and Warner [1], it might be the soup of nutrients, which is more important than an individual nutrient because observational studies with overall dietary practices, such as the Mediterranean and other healthy diets, have been associated with a reduced prevalence of allergies.

Much research has been performed on the positive effects of *breastfeeding* in general, but its effects on the reduction of the development of allergies is underexposed. Chi-Nien Chen and colleagues found, in a study with 6000 children, that exclusive breastfeeding for four to six months was associated with a decreased risk of asthma in children three to six years old, although the effect appeared to diminish at older age [4]. However, in a narrative review by Danielewicz, it becomes clear that outcomes of these kinds of population-based studies are influenced by factors, such as human milk oligosaccharide composition, as well as environmental (allergen exposure, caesarean section), dietary (short fatty acids and introduction of solid food), and immunological factors [5]. As an illustration of this, Macchiaverni and colleagues showed an increased risk of having high levels of IgE and a trend of increased asthma risk in children breastfed by mothers with detectable Der p 1 in human milk, while such an association was not found for Der p 1 in infant mattresses [6].

There exists much evidence for the prevention of food allergies in infants at high risk by *early introduction* of potential allergens, such as peanuts, tree nuts, and eggs, as summarized by Trogen and colleagues [7]. However, further studies are necessary regarding their effects in low-risk patients. As there are multiple potential allergens, which have to be introduced, and which take much time and adherence of parents and patients, the study from the group of Quake is hopeful, as they showed that early introduction of simultaneous mixtures of multiple allergenic foods may be safe and efficacious for preventing food allergy [8]. Although much evidence of the effect of early introduction of peanut and tree nuts on allergy development, less is known about the introduction of cow’s milk, as many children already receive cow’s-milk-based formula much earlier in life. In relation to cow’s milk allergy, Ulfman and colleagues discussed, in a narrative review, that studies have shown that breastfeeding from birth with early introduction of cow’s milk supplementation within the first month of life and continued daily consumption of small amounts without hampering breastfeeding may reduce the risk of developing cow’s milk allergy [9]. However, when the introduction of cow’s milk is interrupted by periods of avoidance, the risk of developing cow’s milk allergy seems to increase.

The use of (partial) *hydrolysed milk formula* to prevent cow’s milk allergy is still being debated, as depicted by Vandenplas and colleagues [10]. Although in vitro and animal studies indicate benefit, there is insufficient evidence to recommend their universal use. Furthermore, hydrolyzed milk formula differs in its degree of hydrolysis and composition (e.g., supplementation with pre- and probiotics), and so much more research has to be performed on their safety and effectivity, but also how they can induce tolerance induction, as shown by Freidl’s et al. [11]. Knowledge on which milk components are key factors in inducing the immune system to a tolerogenic state will enhance the development and application of hydrolyzed milk formula for both treatment and prevention of cow’s milk allergy. Regarding this, the milk allergen micro-array (MAMA), described in this special issue by Garib et al. [12], may be an interesting novel tool to map IgE reactivity to many milk components simultaneously, using a *component resolved diagnostic* approach.

Finally, the potential impact of *early life nutrition and infection* was touched upon by Porbahaie et al. [13], who studied the effect of immune complexes consisting of bovine milk-derived IgG and the RSV pre-F protein. They demonstrated that such immune complexes may induce innate trained immunity, which improves the ability of the innate immune system in relation to TLR stimulation. As such, it may lead to increased innate immune reactivity and immune protection upon infection. Although this work focused on the functional effect of these immune complexes, consuming pasteurized milk during nasal RSV infection may bring these components together and thus influence innate immune reactivity to infectious challenges.

## 3. Concluding Remarks

In this Special Issue, several effects of dietary components on the development of allergy have been described. Not all relevant aspects and dietary approaches have been covered (e.g., microbiota targeted interventions via prebiotics and milk oligosaccharides, although probiotics were discussed), and the main focus turned out to be on allergy, rather than on infection, in this Special Issue. It is important to stress that breastfeeding for the first six months of life is very important for immune development, and it is associated with protection against infections, and it is also dependent on compositions related to allergies. Therefore, exclusive breastfeeding is recommended by WHO for the first six months of life, and other nutrition during this period should only be given if breastfeeding is not possible.

In conclusion, it is clear that dietary modulation of immune function in early life is an important, relevant topic, as well as an important approach, which can help to support immune health in the fist 1000 days of life.
